# Miniature battery-free epidural cortical stimulators

**DOI:** 10.1126/sciadv.adn0858

**Published:** 2024-04-12

**Authors:** Joshua E. Woods, Amanda L. Singer, Fatima Alrashdan, Wendy Tan, Chunfeng Tan, Sunil A. Sheth, Sameer A. Sheth, Jacob T. Robinson

**Affiliations:** ^1^ Department of Electrical and Computer Engineering, Rice University, 6100 Main St, Houston, TX 77005, USA.; ^2^ Motif Neurotech, 2450 Holcombe Blvd, Houston, TX 77021, USA.; ^3^ Applied Physics Program, Rice University, 6100 Main St, Houston, TX 77005, USA.; ^4^ Department of Neurology, UTHealth McGovern Medical School, 6431 Fannin St, Houston, TX 77030, USA.; ^5^ Department of Neurosurgery, Baylor College of Medicine, 1 Baylor Plaza, Houston, TX 77030, USA.; ^6^ Department of Bioengineering, Rice University, 6100 Main St, Houston, TX 77005, USA.; ^7^ Department of Neuroscience, Baylor College of Medicine, 1 Baylor Plaza, Houston, TX 77030, USA.

## Abstract

Miniaturized neuromodulation systems could improve the safety and reduce the invasiveness of bioelectronic neuromodulation. However, as implantable bioelectronic devices are made smaller, it becomes difficult to store enough power for long-term operation in batteries. Here, we present a battery-free epidural cortical stimulator that is only 9 millimeters in width yet can safely receive enough wireless power using magnetoelectric antennas to deliver 14.5-volt stimulation bursts, which enables it to stimulate cortical activity on-demand through the dura. The device has digitally programmable stimulation output and centimeter-scale alignment tolerances when powered by an external transmitter. We demonstrate that this device has enough power and reliability for real-world operation by showing acute motor cortex activation in human patients and reliable chronic motor cortex activation for 30 days in a porcine model. This platform opens the possibility of simple surgical procedures for precise neuromodulation.

## Teaser

Miniature battery-free stimulators fit within the skull and wirelessly stimulate the brain.

## INTRODUCTION

Implantable devices that electrically stimulate the central or peripheral nervous system are increasingly used to treat psychiatric (*1*–*3*), movement (*4*, *5*), and pain disorders (*6*) and for restoration of movement after spinal cord injury (*7*, *8*). Despite demonstrated clinical efficacy, adoption rates for bioelectronic therapies such as deep brain stimulation (DBS) often hover around 5 to 10% (*9*). The leading factors that limit adoption are patient perception of risk, high procedural costs, and long wait lists for the complex procedures (*10*). In general, there is a trade-off in neuromodulatory technologies between the invasiveness and efficacy of stimulation. More invasive devices may be able to target more precisely but suffer from increased real and perceived risk and reduced access due to the complexity of the procedure, which requires specialized surgeons and facilities. For example, the NeuroPace Responsive Neurostimulation and PicoStim systems allow precise access to the brain without long leads in the body but still require large craniotomies and invasive procedures (*11*, *12*). A miniaturized device that could be implanted with minimal risk and a simple procedure but still have precise tissue activation could address many of these limitations.

One method to mitigate procedural risk but maintain tissue contact is through intravascular or epidural placement. Devices placed within the vasculature (*13*, *14*) are able to activate nervous tissue without direct contact thereby simplifying delivery and avoiding damaging the tissue of interest and the direct neural immune response. Intravascular devices, however, have several important limitations: They are often limited to placement near large blood vessels, difficult to explant, and require the patient to stay on antithrombotic medication. For surface targets, placing devices in the epidural space can also allow access to neural tissue without direct contact. Epidural stimulation of the spine has successfully been used for the treatment of pain (*6*) and for movement restoration in spinal cord injury (*7*, *8*). More recently, epidural brain stimulation is being investigated for treatment of major depressive disorder (*1*, *15*), stroke rehabilitation (*16*), pain (*17*), and aphasia (*18*). Although epidural stimulation of the brain requires a small craniotomy, it does not suffer from many of the same limitations as intravascular devices. A major challenge for miniature epidural stimulators is achieving high enough stimulation current amplitudes, which are often more than three times greater than for devices that make direct contact with the target tissue (*19*–*22*) due to the additional distance between the electrodes and excitable tissue. In these cases, small batteries would struggle to provide sufficient power. Practical systems would therefore rely on wireless power transfer (WPT) technologies which are rapidly being developed to allow wireless battery recharging or continuous wireless power supply for these devices (*23*).

Recent advances in materials and electronics are enabling more efficient and robust WPT that can better support implanted bioelectronics with high power demands. Thanks to these advances, there is a growing field of wireless and battery-free neurostimulation technologies including clinical spinal cord stimulation devices (*24*, *25*), injectable neuromuscular stimulation devices (*26*, *27*), and rodent brain stimulation devices (*28*–*30*). We hypothesized that, expanding on these advances, we could create a cortical brain stimulator with sufficient energy to activate human cortical tissue through the dura but still small enough to be implanted into a roughly 14-mm standard burr hole. To meet our millimeter-size requirements, we designed a device that includes no implanted batteries since the battery is the largest volume component of implantable neural stimulators (*31*). As a result, we needed to develop a technology to safely deliver enough wireless power to generate the 2- to 20-mA stimulation currents needed for epidural brain stimulation (*22*) and a corresponding communication system for real-time digital programmability and verification of operation. Here, we demonstrate the first millimeter-scale leadless brain stimulator in a human subject. This Digitally programmable Over-brain Therapeutic (or DOT) is 9 mm across yet is capable of receiving enough energy to stimulate human and large-animal brain activity on-demand through the dura. We use the DOT to apply a form of minimally invasive neuromodulation we refer to as externally powered cortical stimulation (XCS). The device is capable of reliable bidirectional communication with a fully external transmitter. The DOT is sealed in a glass package and maintains reliable operation in freely behaving pigs over the course of 1 month.

## RESULTS

### A miniature, wireless, and battery-free system for epidural cortical stimulation

**Fig. 1.**
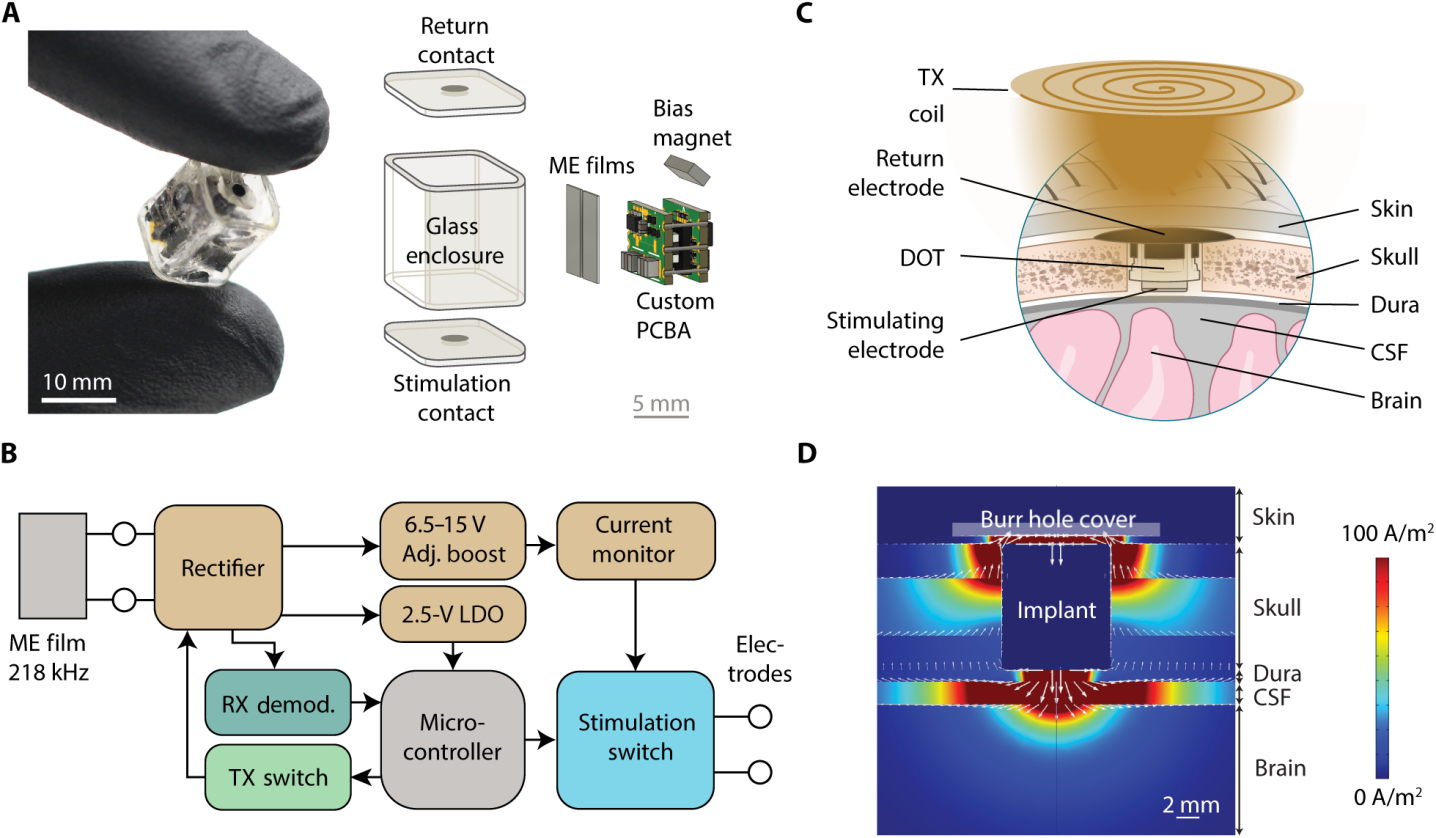
System overview for DOT device. (**A**) Fully packaged DOT held between two gloved fingers and exploded view of the DOT enclosure and internal components. (**B**) Circuit diagram of the PCBA. (**C**) Schematic of the implantation location and surrounding tissue. LDO, low-drop-out regulator. (**D**) COMSOL simulation showing the current density with a pseudo-monopolar electrode configuration. CSF, cerebrospinal fluid.

Recent development of magnetoelectric (ME) wireless power makes it possible to reach the required stimulation compliance for epidural cortical stimulation in millimeter sized, battery-free implants. Our lab has demonstrated up to 56 mW of WPT using ME antennas (*32*) with usable rectified voltages up to 10 V. Briefly, ME antennas are laminates consisting of magnetostrictive and piezoelectric elements that produce power when they vibrate in an alternating magnetic field. To efficiently use this energy and enable 14.5-V compliance across 1-kilohm electrodes, we implemented a circuit using a printed circuit board assembly (PCBA) and completely off-the-shelf available embedded system components (Fig. 1A). Briefly, the circuit includes an efficient power rectification scheme, low-power microcontroller, uplink communication switch, and boost converter with 15-V compliance (Fig. 1B). The board is powered by two 7.5 mm by 3 mm ME antennas connected in parallel with each other. These antennas operate at a resonant frequency of 218 kHz. We then designed a custom glass enclosure that includes a square glass tube—chosen to match the geometry of the internal components while minimizing the volume of the device—and two caps patterned with sputtered iridium oxide electrodes connected to the inner device using through-glass via hermetic feedthroughs (Schott HermeS wafers). These caps are sealed to the tube with the circuit and antennas inside using a medical grade epoxy (MED-301-2, Epo-Tek). The completely packaged device is 9 mm by 9 mm by 11 mm.

To power and communicate with the DOT, we engineered a magnetic coil and driver system that transmits power and data to the implant. The transmitter for this application consists of an H-Bridge driver powering an LC resonant 6 cm in diameter three-layer pancake transmitter (TX) coil. This coil can be optimized for different desired operating depths and alignment tolerances if desired. The transmitter generates an alternating magnetic field at the resonant frequency of the ME antennas used in the device (218 kHz), a relatively low frequency that is able to pass nearly losslessly through biological tissue (*33*). To our knowledge, this frequency is not in a protected band and is relatively close to the 175-kHz frequency band used in medical devices for years (*34*). The resonant frequency depends on geometry, which can be precisely tuned with the laser cutting method used (see Materials and Methods). The transmitter voltage is adjusted to output a maximum of 7-mT alternating magnetic field, which is within the safety limits for absorption at this frequency (*35*). A receiver (RX) coil consisting of differentially paired coils on a PCB is placed between the TX coil and the scalp for uplink communication.

The system as intended for chronic implantation is shown in Fig. 1C. The DOT is surrounded by a silicone sleeve and placed within a 14-mm burr hole in the skull with the bottom electrode in contact with intact dura. Preliminary studies and simulation show that current spreads farther into the brain when the return is placed on top of the device (Fig. 1D) rather than next to the stimulating electrode on the bottom. Therefore, we cover the device with a biocompatible plastic [polyetheretherketone (PEEK)] cover that has a hole in the top to allow for fluid and interstitial tissue and fluid to make contact with the top electrode. We also used a stainless steel conductive mesh to further ensure that the top electrode is electrically connected to the inner surface of the tissue in the case of insufficient conduction due to biological fluids. We expect that the infection risk related to this procedure and device will be quite favorable relative to other cranial neurosurgical procedures due to the small incision, short procedure time, and lack of need for reoperation. The system is then powered and controlled by placing the TX coil on the surface of the skin above the device.

### High voltage WPT and digital bidirectional communication

**Fig. 2.**
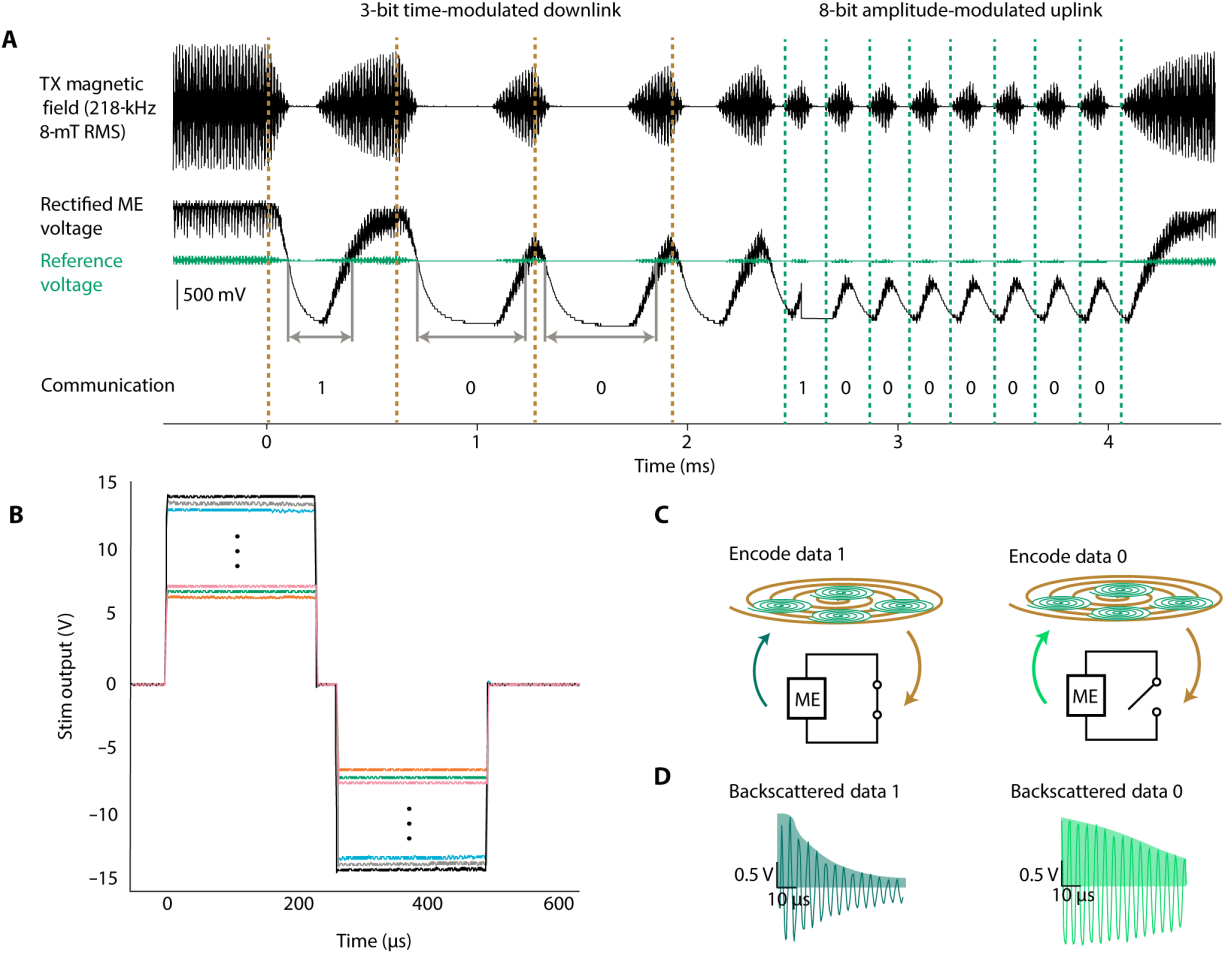
Wireless communication protocol and device outputs. (**A**) Communication protocol for the DOT showing downlink of message 0b100 and uplink of message 0b10000000. (**B**) Digitally programmable stimulation output pulses showing 250-mV increments between settings. (**C**) Schematic showing how digital data are encoded by passive ME backscatter. Bits 1 and 0 are encoded by switching the nodes of the ME film between short (data 1) and open (data 0). The films are excited by the transceiver field, and the resulting ringdown field is measured from differentially paired pickup coils while the excitation field is off. (**D**) Examples of the measured ringdown field during a 1 with a time constant of 40 μs (dark green) and 0 bit with a time constant of 91 μs (light green). Data are decoded as an integration of the positive half of the signal (shaded).

The device is designed to harvest the energy from the ME films and digitally control programmable high-power stimulation. To minimize size, complexity, and power demands of the implant, we use the ME material for both downlink and uplink communication (*36*). Here, we implement a communication scheme where each 3.4-ms duration message has a downlink section containing 3 to 5 bits (1.8- to 3.0-ms duration total) and an uplink section containing 8 bits (1.6-ms duration total) (Fig. 2A).

Downlink communication is encoded in the duration of time that the magnetic field is off relative to the time that it is on. In a data 0 bit, the field is off for 200 μs and on for 100 μs; in a data 1 bit, the field is off for 100 μs and on for 200 μs. This is decoded on the implant by comparing the unsmoothed rectified voltage with the capacitor smoothed supply voltage across a diode. In this way, when the field is off, the rectified voltage drops below the supply voltage, and when it is turned back on, the rectified voltage exceeds the supply voltage. We obtain 5-bit control over the amplitude by following the 3-bit amplitude priming command with a 5-bit digital amplitude packet. For these studies, we programmed the device to output 250 and 500-μs pulse width stimulation pulses between ±6.75 V and ±14.5 V in 250-mV increments (Fig. 2B). With the measured in vivo impedances of approximately 1 kilohm, this corresponds to stimulation pulses of ~6.75 to 14.5 mA of current.

To receive real-time feedback, after the downlink portion of each message is an 8-bit uplink portion. To receive real-time diagnostic information from the implant, we implemented uplink communication using on/off keying of the backscattered magnetic field, which consumes almost no energy from the implant. Briefly, we turn on the transmitter field for 100 μs to excite the ME film resonance mode and then turn it off for 100 μs to record the residual magnetic field generated as the ME resonance decays (also known as “ring down”). A switch on board the implant modulates the amplitude of this reflected signal (Fig. 2C). If the output of the ME film is electrically connected to our stimulation circuit, then the ring down will be long, but if the output of the film is electrically shorted, then the ring down is much shorter (Fig. 2D). The backscattered magnetic field voltage from the ME film is received using differentially paired coils printed on a custom PCB and aligned with the transmitter coil. In this way, the large magnitude excitation field is not amplified, but the small amplitude backscattered field is. A custom analog front end circuit consisting of a preamplifier, variable gain amplifier, and active low-pass filter filters and amplifies the signal. An analog envelope detector smooths the output, and an onboard microcontroller (NXP LPC54605) samples this signal during the ringdown to decode the data in real time. To set the threshold between 0 and 1 during use, a calibration signal consisting of sequential bits 01010101 is used periodically.

**Fig. 3.**
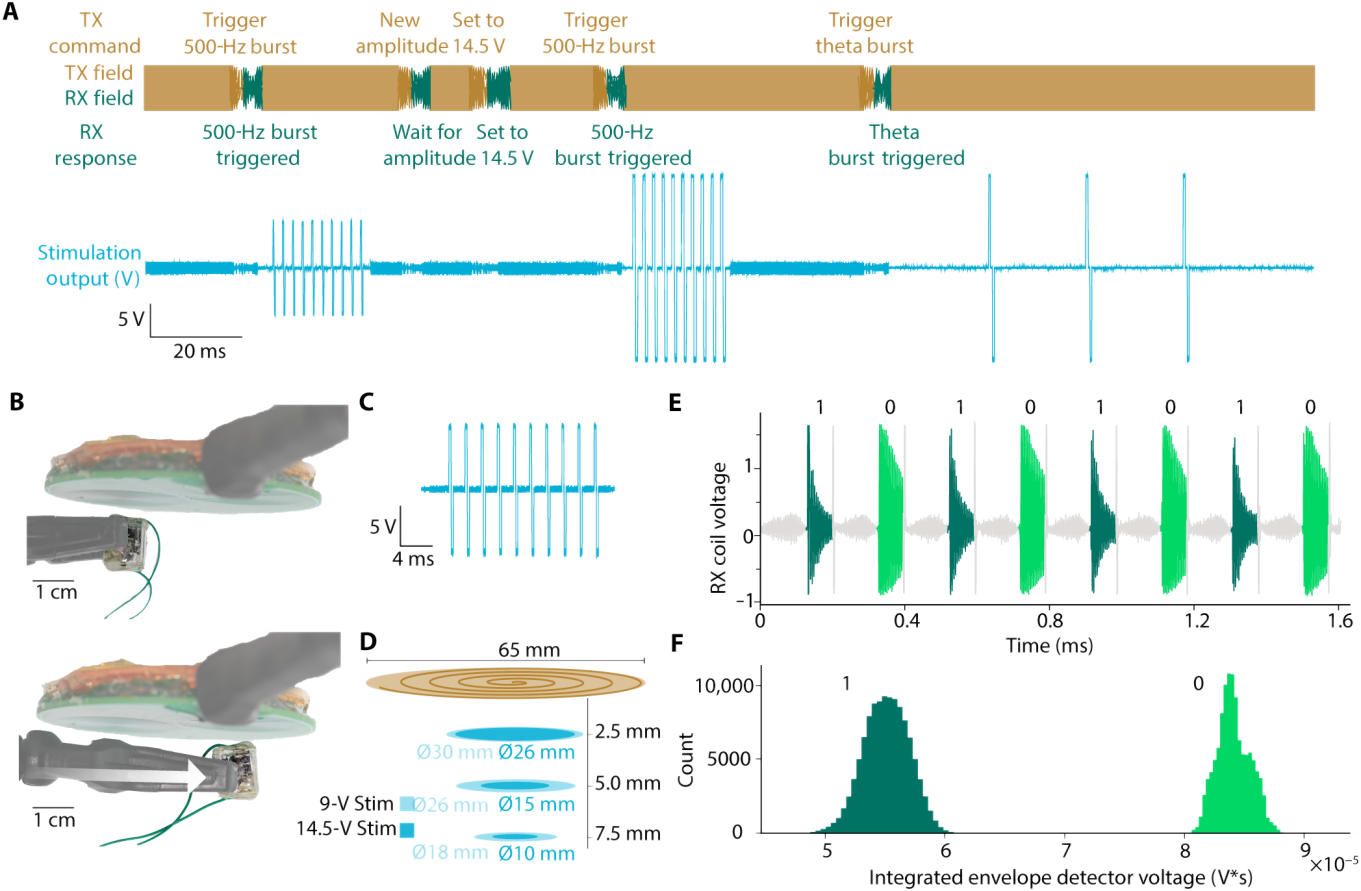
Characterization results for DOT WPT and communication. (**A**) Example experimental sequence of the transceiver field and implant stimulation outputs. The transmitter field (orange) provides power during the charging phases, followed by programming phases where digital downlink data (dark orange) are transmitted to the device and uplink data (green) are returned. Here, the device was programmed to have different output voltages, stimulate with 10-pulse 500-Hz pulse trains, and stimulate with 3-pulse 50-Hz pulse trains. The output voltage generated by the stimulator (blue) shows the different patterns of stimulation generated based on the transmitted instructions. (**B**) Experimental images showing two positions where the device is able to output biphasic 9-V, 10-pulse, 500-Hz pulse trains across a 1-kilohm resistor with a field strength of 7 mT at the coil surface and the corresponding simulation output. (**C**) Example biphasic 9-V, 10-pulse, 500-Hz pulse train from the device. (**D**) Plot showing experimentally determined locations the device is able to output biphasic 9- or 14.5-V, 10-pulse, 500-Hz pulse trains across a 1-kilohm resistor at different distances from the transceiver coils with a field strength of 7 mT at the coil surface. (**E**) A sequence of 1 and 0 bits used to calibrate the threshold for differentiating the two bits. This calibration sequence is used on demand to account for changes in implant positioning relative to the transceiver. (**F**) A histogram showing the integrated voltage of the ringdown signal for bits 1 and 0 with 223,888 sampled bits with the implant at a distance of 1 cm from the transceiver coils. Bits 1 and 0 are very well separated and easy to differentiate from the receiver.

Stimulation parameters and other instructions can be controlled using the downlink portion of the message and status updates, and current measurements can be transmitted by the device using the uplink portion of the message. An example sequence is shown in Fig. 3A where the device is sequentially programmed to (i) apply a 500-Hz pulse train at 9-V amplitude, (ii) increase the amplitude to 14.5 V, (iii) apply a 500-Hz pulse train at 14.5 V, and (iv) apply a lower-frequency pulse train at 14.5 V.

The misalignment tolerance of the power and data transfer technology enables reliable wireless data and power transmission in uncontrolled environments like the operating room. Good misalignment tolerance is a known quality of ME power transmission (*37*), but the exact degree of tolerance depends on the entire system configuration. To quantify the alignment tolerance for our system, intended for XCS, we adjusted the transceiver to produce a 7-mT magnetic field [within the safety limits at this frequency (*35*)] at the surface of the coil and measured the locations at which the device received enough power to produce 10 250-μs per phase stimulation pulses at 500 Hz across a 1-kilohm resistance (Fig. 3, B and C). When outputting 9-V biphasic stimulation pulses, the device operated across a diameter of 1.8 cm (2.5-cm^2^ area) at the center of the transceiver coil at a distance of 7.5 mm from the coil (measured to the center of the top of the device). When outputting maximum compliance, 14.5-V biphasic stimulation pulses the device operated across a diameter of 1 cm (0.78-cm^2^ area) at a distance of 7.5 mm from the surface of the coil (Fig. 3D). These distances compare favorably to expected implantation depth based on reported 5.8-mm average human scalp thickness (*38*). In the calibration sequence (Fig. 3E), bits 0 and 1 are easily differentiable at distance of 1 cm from the transceiver coils (Fig. 3F).

### Enough wireless power to stimulate the human motor cortex from above the dura

**Fig. 4.**
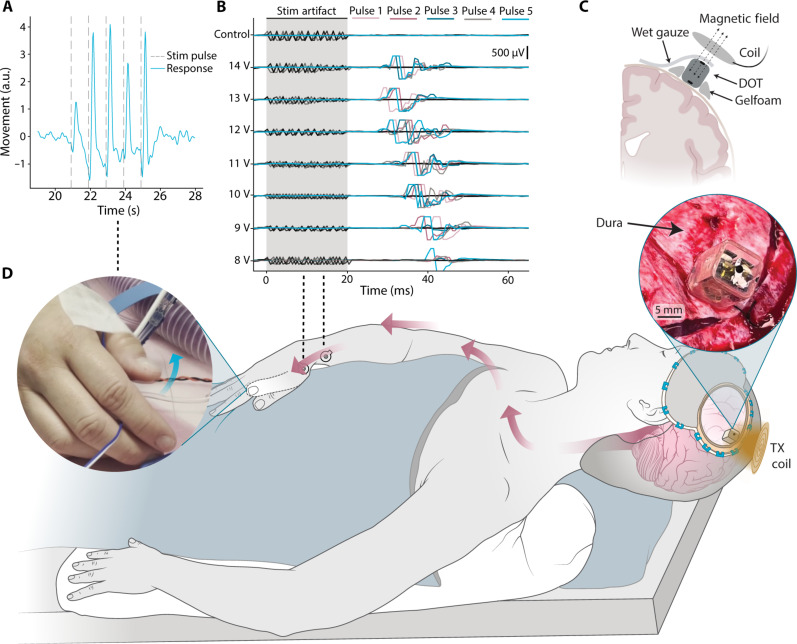
Intraoperative demonstration of epidural cortical stimulation with a millimeter-sized battery-free device. (**A**) Displacement of the thumb extracted from videos during epidural cortical stimulation shows movement in response to each stimulation pulse train applied by the DOT (vertical dashed lines). (**B**) EMG traces recorded from 5 pulses each in the APB-ADM muscle groups during stimulation at varying amplitudes. (**C**) Schematic of the intraoperative placement of the device. Saline wetted gauze is used to make electrical connection with the top electrode. (**D**) A schematic of the intraoperative human studies, where we placed the device above the dura on the motor cortex and activated it with the transmitter coil. Stimulation resulted in contralateral thumb movement. The left inset shows a frame of the movie (movie S2) used to analyze the thumb movement, and the right inset shows a photograph of the DOT placed over the dura. a.u., arbitrary units.

Intraoperative testing during a neurosurgical procedure demonstrated that the DOT could stimulate a motor response when placed directly on the motor cortex with enough energy to drive a hand contraction (fig. S1 and movie S1). We decided to perform human studies because of the differences in the anatomy of the human brain compared to the porcine large animal model, which can affect stimulation thresholds (*22*, *39*). In this study, a patient was undergoing a procedure for tumor resection that required a craniotomy to expose the motor cortex. As part of this procedure, the surgical team mapped the motor cortex using standard electrophysiological procedures to identify the region of motor cortex that, when stimulated, produced a hand contraction (see Materials and Methods). We then provided the DOT and transmitter to the surgical team who placed the DOT on the identified region of the target motor cortex, connected the top electrode to the surrounding tissue with saline wetted gauze, and placed the wireless transmitter above the DOT (Fig. 4C). After programming the DOT to produce 250-μs pulse width, 500-Hz, 10-pulse trains at 1-Hz frequency and 14.5-V biphasic amplitude, we observed and analyzed a video recording of 1-Hz contraction in the hand confirming that we can activate substantial regions of the motor cortex on-demand.

A second intraoperative study shows that the DOT is also able to stimulate similar motor responses when placed above the dura (Fig. 1 and movie S2). In this study, we followed the same protocol as our first patient, but once we identified the target in the motor cortex, we laid the dura back on top of the brain and placed the DOT over the dura (Fig. 4D, right inset).

With the dura in place, the DOT elicited visible thumb movements above 10-V stimulation amplitude (Fig. 4D, left inset). Analysis of video recordings with DeepLabCut analysis software (*40*) shows clearly distinguishable muscle responses with each stimulation pulse (Fig. 4A). Electromyography (EMG) responses in the APB-ADM (Abductor pollicis brevis, Abductor digiti minimi) muscle groups were recorded at amplitudes as low as 8 V, as the stimulation intensity increased, the latency between stimulation and response decreased and the number of elicited movements increased (Fig. 4B). This precise localization of stimulation response demonstrates the fine precision of the DOT’s activation area. This shows that the high voltage compliance of the DOT is necessary and adequate to activate tissue in the human motor cortex from above the dura due to the increased distance and additional interposing tissue.

### Robust operation over 30 days in freely behaving pigs

Our intraoperative studies confirmed that the DOT was powerful enough to stimulate the motor cortex through the dura, but we also wanted to confirm that our device would be able to provide this level of stimulation over time if it was chronically implanted. For this study, we used a porcine model, which is commonly used because the porcine brain anatomical structure (*41*) and dural thickness (*42*) are most consistent with human anatomy. In this preparation, we tested the DOT’s ability to stimulate through intact dura and the impact on tissue response over time. We also tested our ability to power and communicate with the device in a freely behaving large animal model.

**Fig. 5.**
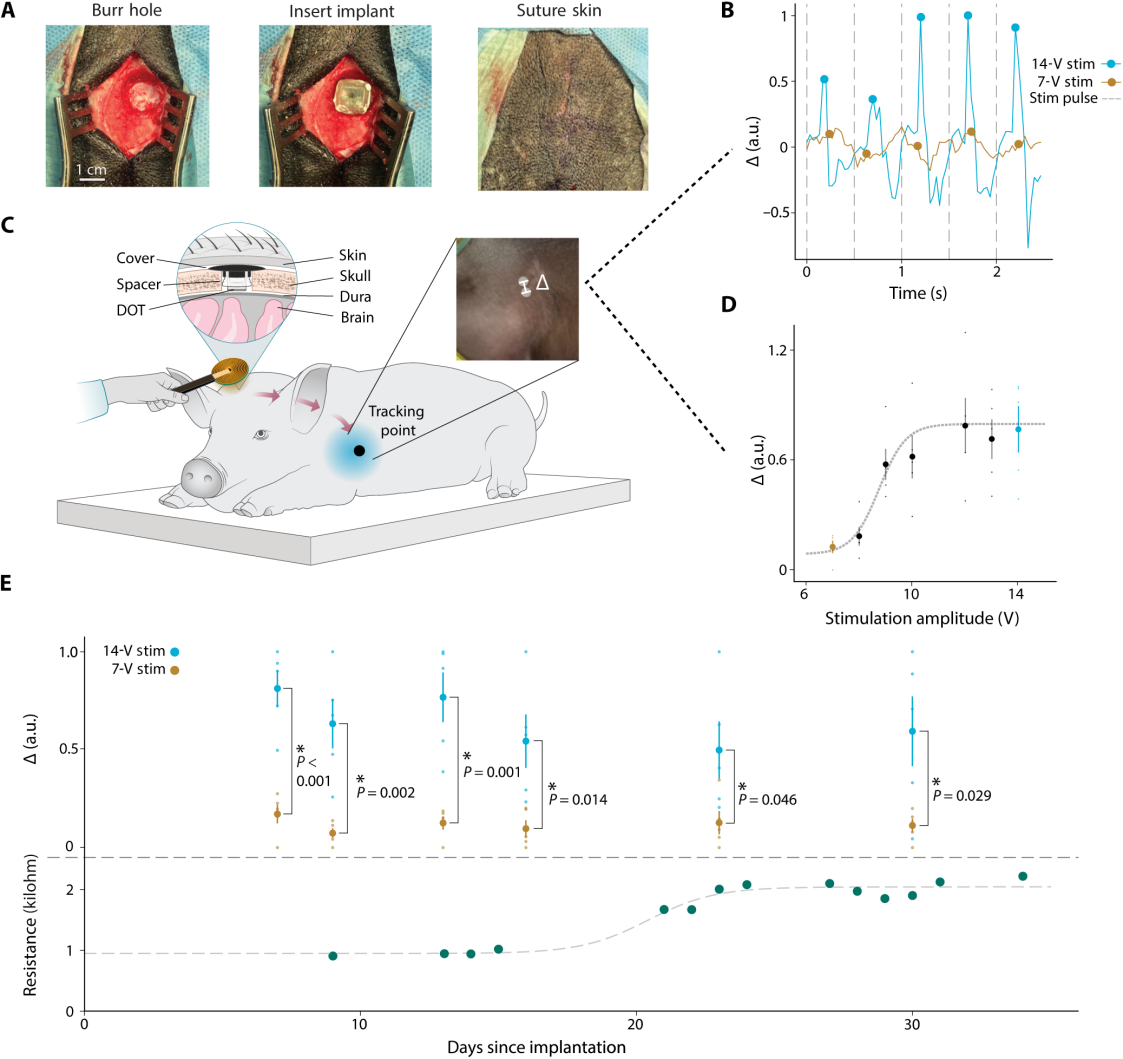
Thirty days of epidural cortical stimulation in a large animal model. (**A**) Surgical photos of the DOT implantation procedure. (**B**) Forelimb movement in response to stimulation extracted from experimental videos using DeepLabCut. The peaks of the movement in the 350 ms following stimulation at 7-V stimulation amplitude (yellow) and 14-V stimulation amplitude (blue) are marked with circles. (**C**) Schematic of device testing during chronic implantation in a porcine model. The schematic is reflected for visualization. Left inset: A cross section of the DOT implant above the dura and below the skin of the pig. Right inset: An example frame from the video used to quantify forelimb movement. (**D**) Mean of five movement peaks extracted from a video with increasing stimulation amplitude from 7 to 14 V, and error bars represent SE. Dotted line shows a sigmoidal fit with an approximate motor threshold at 10 V. The 7-V (yellow dot) response is well below the motor threshold which we use as the noise floor, and the 14-V (blue dot) response shows much higher displacement, indicative of movement in response to stimulation. (**E**) Top: Mean of the movement peak amplitudes extracted from videos over the entire implantation period (blue dots). Average movement amplitude of 5 pulses of 14-V stimulation (orange dots), average movement amplitude of five samples at 7-V stimulation. Error bars represent SE. On all days tested, we see significantly more motor evoked response from 14-V stimulation compared to 7-V stimulation (independent samples *t* test, *P* < 0.05). Bottom: Resistance as measured with ME backscatter communication over the entire implantation period. An increase from ~1 to ~2.25 kilohm was seen between days 15 and 25 but leveled off following day 25. Dotted line shows a sigmoidal fit.

The implantation surgery took approximately 30 min and involved no contact with the brain (Fig. 5A). Briefly, we exposed the skull and drilled a 14-mm diameter burr hole over the motor cortex, exposing the dura beneath (see Materials and Methods). We then assembled the implant with a protective silicone spacer, placed it into the burr hole, and secured it with a plastic (PEEK) burr hole cover to protect the implant from external damage. A schematic of the fully implanted system and testing protocol is shown in Fig. 5C. This simple method was sufficient to protect and secure the implants, with none of them being mechanically damaged for the lifetime of our chronic study.

When implanted in pigs for 20 to 35 days above the dura over the motor cortex, the DOT was able to consistently drive motor stimulation. We observed movement in response to stimulation over the entire chronic experiment in *n* = 3 animals (Fig. 5, B and D, and fig. S2). We saw no evidence of activation of subcutaneous pain receptors at the dura or scalp since the pigs often slept through our stimulation experiments; however, only chronic human studies will allow us to reliably assess whether stimulation produces any form of noticeable sensory percept. We also used a sham implant in some animals to compare the pathology over time (see Materials and Methods and table S1). Movement amplitudes following stimulation were extracted using video analysis tools when possible. The results for all 30 days are shown in Fig. 5E (top) normalized by the minimum and maximum amplitudes from each day. We also regularly interrogated the device using our ME backscatter uplink (in *n* = 1 animal) to record the impedance of the DOT electrodes. Between days 15 and 25, we observed an increase in resistance (Fig. 5E, bottom) similar to that seen with DBS electrode impedance (*43*). Between these days, we noted a decrease in movement amplitude at maximum stimulation values that correlated with the increase in resistance. After 25 days, the resistance stabilized at about 2.25 kilohm, which could be due to anatomical changes in the animal including thickening of the dura or tissue regrowth in the burr hole cavity. These are known foreign body responses that can occur in subdural and epidural implants but do not present a safety concern (*44*). The fact that the DOT continued to deliver effective brain stimulation despite this increase in impedance supports its effectiveness as a chronic therapy even in the presence of foreign body responses.

One potential application for an implantable cortical stimulator would be neuromodulation for psychiatric conditions similar to transcranial magnetic stimulation (TMS). Repetitive TMS (rTMS) is delivered in a clinic using pulsed magnetic fields in excess of one 1 T to stimulate the brain. rTMS has shown clinical efficacy in treating conditions like treatment resistant depression, obsessive compulsive disorder, post-traumatic stress disorders, and other psychiatric disorders but requires frequent visits to a clinic. Because neuromodulation for psychiatric conditions is a potential application for the DOT, we approximated the therapeutic stimulation doses that one would receive in a TMS-like therapy. Following this study, we observed no serious pathology in the stimulated brain areas, suggesting that the DOT stimulation may provide a safe analog to TMS therapies but without the need for frequent visits to a clinic. For these studies, we chose to approximate the intermittent theta burst stimulation (iTBS) paradigm used by TMS to stimulate cortical areas with specifically timed pulses over the course of 3 min. This waveform consists of 50-Hz bursts of 3 pulses repeated at a rate of 5 Hz and is traditionally repeated for 2 s at 10-s intervals. Studies have shown that variations of this stimulation protocol repeated 5 days a week for 6 weeks provide effective therapy for patients with treatment resistant depression (*45*, *46*). The total dose received in such a therapy amounts to a total of 60 to 450 min of stimulation and a total of 12,000 to 90,000 pulses (*47*, *48*). In testing the safety of DOT stimulation, we applied direct electrical stimulation of an iTBS-like waveform with 250 μs per phase biphasic pulses for a total of 300 min (54,300 pulses). In two other animals, we stimulated for a total of 170 and 200 min of iTBS-like stimulation. We then explanted the devices and survived the animals for 7 to 10 days before sacrificing the animal to analyze the brain and dura. Pathology reports of one animal post-explantation showed no difference between the brain or dura beneath the stimulator when compared to a sham implant of the same size and shape in a separate animal (fig. S3). In some animals, we found minor inflammatory responses to both the active and sham implants, suggesting animal-to-animal variability in their foreign body responses, but even in the case of this foreign body response, the DOT could still generate effective motor cortex stimulation, suggesting that this response would not limit its ability to provide effective neuromodulation therapies.

## DISCUSSION

The data presented here show reliable activation of the motor cortex with a miniature wireless epidural device. To our knowledge, this is the smallest implantable brain stimulator demonstrated acutely in a human subject, which suggests that this type of battery-free bioelectronic technology could serve as a therapeutic platform with reduced surgical risk. For this work, we chose to use ME materials for power transfer due to the high power density and large misalignment tolerances. It is possible that alternative power solutions like emerging magnetic resonant power schemes could achieve similar performance; however, the scaling of ME power transfer is superior to magnetic induction so ME power is likely to be the preferred solution as devices are further miniaturized.

This was a proof-of-concept system, and there are many aspects of the work that should be considered for future application. First, voltage-controlled stimulation was used to minimize system complexity and number of off-the-shelf circuit components. As a therapeutic implant, future versions of the device should incorporate current-controlled, charge-balanced stimulation to ensure safe chronic activation of tissue. A detailed analysis of the stimulation and return electrode size and placement should also be performed to optimize the stimulation parameters, voltage compliance requirements, and patient comfort. In addition, the packaging should be made hermetic to last several years and should be fully verified for biocompatibility, durability, and reliability. Last, the low data rate obtained in this resonant system should be improved or changed to allow higher bandwidth communication and enable device authentication, error checking, and potentially encryption.

When miniature battery-free implants that can effectively stimulate neural activity from the epidural space reach the clinic, they will create an architecture for neuromodulation systems that will enable new paradigms for clinical therapy. For example, episodic neuromodulation that is known to be effective for TMS may one day be applied safely at home with a precisely targeted implant combined with a wearable headset that need only align approximately with the neuromodulation target. The simplicity, safety, and minimal management burden of XCS in this episodic use case would allow patients to avoid the maintenance checks required for implanted batteries and leads, especially rechargeable batteries that have to be periodically charged to maintain battery health. The system could also be expanded by increasing the number of wirelessly powered implantable devices. For example, with multiple implants that can be triggered with precise temporal patterns, one could develop more accurate ways to modulate the brain states that are believed to be associated with mood and memory (*49*) or activate distributed locations across the spinal cord for movement restoration or pain treatment. To modulate brain states with precise timing, integrating neural sensing or other sensing capabilities with these devices could allow closed-loop control of brain activity.

## MATERIALS AND METHODS

### PCBA design

The custom PCBA is designed to harvest energy from the ME films and digitally control the output stimulation. It consists of a Schottky diode bridge rectifier, storage capacitors, low-dropout regulator, boost converter (LTC3129), current sense amplifier (INA186), output switch (DG636), and Microcontroller (NXP KL15). Stimulation amplitude is controlled by setting the boost converter voltage using the digital to analog converter on-board the microcontroller. Output stimulation is switched on and off using the output switch. Charge-balanced output is maintained by stimulating with the two electrodes sequentially. First, the bottom electrode stimulates with the stimulating voltage, while the top electrode is connected to ground, and then the top electrode stimulates while the bottom electrode is connected to ground. Output resistance is approximately measured using the current sense amplifier. The amplifier is set to monitor the supply current of the stimulation output switch. By sampling this current during stimulation output, with the knowledge of the programmed voltage, an approximation of the output resistance can be calculated. This was verified using varying stimulation voltages and load resistances to be accurate across the expected operation region.

### DOT assembly

The DOT consists of four main components: the PCB assembly, two 7.5 mm by 3 mm ME films, a bias magnet, and three-part glass enclosure. Briefly, ME films were manufactured by epoxying (M-Bond 43-B) PZT (Piezo Systems PZT 5H PSI-5H4E) and Metglas (2605SA1, Metglas Inc.) together into a three-layer laminate with Metglas on both sides of the PZT and then cutting the laminate with a femtosecond laser (One Five Origami XP, NKT Photonics). Once cut, films were tested for functionality in a 218-kHz alternating magnetic field, and those with a saturation voltage above 25-V peak to peak were kept for use in devices. The custom PCB was designed and manufactured in separate panels by a commercial manufacturer (PCBway) and then assembled by hand in the laboratory. For each device, the two panels were joined together using seven 3-mm lengths of uninsulated wire soldered between castellated vias on the sides of the panels. Once joined, the devices were tested for basic functionality using test firmware. Completely functional PCBAs were flashed with final firmware, and then the ME films were attached in parallel to the top panel with vertically placed 0-ohm resistors and conductive epoxy. Once films were attached, the devices were tested in a magnetic field with an external bias magnet to ensure that the films provided enough power for stimulation and other functions. Then, a 1 mm by 2 mm by 3.5 mm neodymium bias magnet was fixed in place on the assembly to allow for a wider range of motion than can be accomplished with a fixed external bias. The bias magnet is necessary for the ME films to operate in optimal length mode resonance (*50*). The magnetic properties of this bias magnet are expected to produce strong magnetic resonance imaging (MRI) artifacts but to remain MRI safe. This labeling is comparable to DBS devices. Once operation was again verified, the test pins were cut off of the PCBA using a sanding disk attachment on a benchtop lathe.

The glass enclosure consisted of three separate parts of borosilicate glass, a rounded square tube (F&D Glass) and two caps with an electrode in the center. The caps were patterned and fabricated from a custom glass HermeS wafer (Schott), which was fabricated with hermetically sealed tungsten vias. The use of this Schott HermeS technology allows the potential for future addition of more electrode contacts. This wafer was masked with polyimide tape to define the placement of the circular electrodes (each 1.5 mm in diameter). The wafer was then sputtered with a coating of 10-nm Ti, 100-nm Pt, 10-nm Ti, and 300-nm IrOx stack (AJA ATC Orion Sputter System) as described by Lycke *et al.* (*51*). After sputtering, the wafer was laser cut into rounded square caps (Universal X-660 Laser Cutter). We chose IrOx because of its previously demonstrated high charge storage capacity and biocompatibility in long-term studies (*51*); however, this material is not yet commonly used in US Food and Drug Administration (FDA)–approved devices. Should IrOx be used in a future commercial human device, it would need to pass biocompatibility and toxicity studies.

To assemble the final device, the bottom cap of the glass enclosure was attached with medical grade epoxy (Epo-Tek MED-301-2) to a 1-cm section of glass tube. Since the intention of this work was short-term proof of concept, to save cost and time, fully hermetic encapsulation was not used, but we expect that hermetic glass enclosures could be assembled by laser welding the glass as has been shown in other FDA-approved glass packages (*52*). The assembly with films was then placed inside the glass tube and stimulation output connected with conductive epoxy to the sputtered electrode on the bottom cap. This second stimulation output was connected with conductive epoxy and a ~1-cm insulated wire to the electrode on the top cap. The top cap was then sealed with medical grade epoxy to complete the enclosure. For chronic and intraoperative studies, the device was sterilized with a 12-hour ethylene oxide sterilization cycle.

### Stimulation electrode testing

As a preliminary test to characterize the durability of the contact electrodes, we performed a benchtop test in phosphate-buffered saline (PBS) with two test devices. Two IrOx electrodes were sputtered 4.5 mm apart on a glass cap and submerged in PBS in a 37°C incubator. The devices were connected to a stimulator programmed to produce 250-μs pulse width, 500-Hz, 6-pulse trains at 2-Hz frequency and 15-V biphasic amplitude, corresponding to roughly 1 million pulses per day. The impedance between the two contacts in saline at 1 kHz was measured daily for 22 days. Over the entire experiment, corresponding to more than 22 million pulses, the devices maintained an impedance between 250 and 400 ohm (fig. S4), indicating that the electrodes did not suffer notable degradation. The roughly 22 million pulses tested here is significantly larger than the roughly 60,000 pulses that are required for therapies like TMS for depression (*46*).

### DOT characterization

Fully assembled DOT devices were tested to characterize operational performance. To measure the separability of 0 and 1 data bits, a fully assembled implant was tested with the calibration sequence. The implant was placed 1 cm above the paired transmitter and receiver coils and powered with a 7-mT magnetic field. The transceiver was programmed to send, receive, and print raw output values for 8-bit calibration signals at a rate of 50 Hz. A total of 223,888 bits were sampled and plotted with a histogram to show separability. No data points were discarded as outliers.

To measure the alignment tolerance of the system for WPT, wires were connected to the stimulation electrodes of a fully assembled implant, connected across a 1-kilohm resistor, and monitored with a 1-megaohm impedance oscilloscope. The transceiver was adjusted to produce a 7-mT magnetic field at the surface. To produce a magnetic field of 7 mT, the transmitter consumes 18 W of peak power but is only required to be operating in this state for approximately 500 ms to deliver a maximum amplitude pulse train. The system efficiency at various distances has been characterized previously (*35*) for a geometry that we expect will be similar to a wearable transmitter. This efficiency combined with the average power requirements for a given therapy is design considerations for a battery powered wearable. Figure 3 shows a characterization of the operating range within the Institute of Electrical and Electronics Engineers safety limits. At each height, the device was repeatedly triggered to output stimulation at the desired voltage and moved along a linear axis from the center of the coil until the last pulse of the 10-pulse train dropped 200 mV below the programmed amplitude, and this was marked as the operational region. Rotational symmetry is assumed on the basis of the symmetric geometry of the transceiver system.

### Intraoperative human testing

Both studies took place during tumor resection surgeries at Baylor St Luke’s Medical Center under Institutional Review Board approval and informed patient consent. Before using the DOT, the patient was placed under anesthesia and had the surgical site prepped, and the motor cortex was exposed via a craniotomy. The location of the primary motor cortex was determined by locating the central sulcus with median nerve stimulation and electrocorticogram recording to determine the location where phase reversal occurred. The specific cortical targets within the primary motor cortex to activate with the DOT were determined from motor mapping with probe stimulator and EMG monitoring (Cadwell IOMAX) by a neuromonitoring technician on site. In the first intraoperative study, the DOT was placed on the right motor cortex, activating muscles in the left hand, while in the second patient, the DOT was placed on the left motor cortex, activating muscles in the right hand.

The physician placed the device above the brain target and used gelfoam to secure it on the cortical surface. To make electrical contact between the top electrode and the tissue (as we would expect in an actual implantation scheme), the surgeon placed a piece of saline wetted gauze over the top of the device to the surface of the dura. The surgeon then placed the transmitter coil above the implant while we powered and commanded the coil to wirelessly power the implant via a computer and elicited a motor response.

Video recordings were taken of the patient’s hand during stimulation with audio cues for stimulation timing. In post-analysis, the audio cues were used to find the approximate timing of stimulation pulses. The videos were then analyzed with DeepLabCut by marking a stationary point in the scene and features on the hand and fingers. The distance between the stationary point and the hand was measured over time, and the signal was bandpass-filtered between 0.5 and 3 Hz since the response to stimulation was at 1 Hz.

### In vivo porcine model

The animal procedures were conducted in accordance with the rules of the Institutional Animal Care and Use Committee (protocol no. AWC-22-0023). The results presented in this work contain results from four cohorts of pigs (two pigs per cohort, for a total of *n* = 8 animals and *n* = 10 implants; see table S1). The first two cohorts provided feasibility insight into the surgical procedure, animal handling, and electrode design. In these two cohorts, all implants were functional when implanted, and the animals followed the same general chronic procedure outlined here, but no chronic stimulation was applied. The outcome of these two early cohorts resulted in changes to implantation procedure (including a burr hole cover) and changes from a bipolar (two electrodes on the bottom of the implant) to a pseudo-monopolar electrode (electrodes on the top and bottom of the implant) design for more effective tissue stimulation (*53*). All of these changes were implemented for cohorts 3 and 4.

In the third cohort, one sham animal received no stimulation, while the other received stimulation. In the fourth cohort, each animal was implanted with two implants-one active implant that provided stimulation to the left motor cortex and one sham implant over the right motor cortex.

Each study took place in six distinct parts: (i) pre-implantation, (ii) surgical implantation, (iii) daily testing, (iv) surgical explantation, (v) terminal procedure and euthanasia, and (vi) tissue harvest.

### Pre-implantation conditioning/acclimation

For 1 to 2 hours a day, we sat in the animal cage and calmly encouraged the animals to approach, lay down, and allow us to touch their head, face, and ears and introduced a testing coil. By the time of implantation, all animals behaved normally around us.

### Surgical implantation

On the day of implantation, veterinary staff prepared the animals and monitored electrocardiogram, respiration rate, end-tidal carbon dioxide, oxygen saturation, rectal temperature, and jaw tone. We arrived and completed surgical implantation of the device, in approximately 30 to 60 min per animal, depending on whether any intraoperative testing was performed.

The implant procedure involved placing the DOT device flush with the skull and atop the dura via a typical neurosurgical burr hole procedure. Briefly, hair was shaved and a small incision was made above the motor cortex, midline, and parallel to the eyes, ~4 cm. A surgical window of 4 cm by 5 cm was cleaned and prepared for cranial burr access. We then drilled a cylindrical burr hole of approximately 14-mm outer diameter (Medtronic Midas Rex drill) down to the dura without puncturing it.

During this procedure, in the third cohort tested, one animal was first anesthetized with isoflurane and then weaned onto ketamine to verify the implant location with a motor response. Previous work has shown that any lingering isoflurane will block the motor response that can be generated from epidural cortical stimulation (*54*). A Cadwell IOMAX and disposable monopolar stimulator probe (Neuroradium Inc.) verified the anatomical target location and stimulation amplitude above the motor cortex. Disposable subdermal needle electrodes (Neuroradium Inc.) were placed on the forelimb of porcine subjects to record EMG activity during this stimulation. Once targeting above the motor cortex was confirmed, the DOT and accessories were assembled and implanted in the burr hole space (fig. S2) and driven to an electrical stimulation output sufficient to elicit a similar motor output to that observed with the Cadwell IOMAX system. Last, the surgical site was sutured closed and the animal went into recovery.

In the fourth cohort, no intraoperative testing results were used for targeting or stimulation verification. The DOT was simply implanted in the same area successfully used in the previous cohort.

#### Daily testing

Extensive veterinary monitoring occurred for 5 to 7 days taking note of attitude, incision health, and appetite and application of pain medication. To test the animals following surgery, we entered the pen and spent time feeding and calming the animals until they laid down for rest. Then, the external magnetic field driver was placed above the implant site.

After that, therapy workflow included (i) running a motor stimulation waveform paradigm and observing any motor output in the right shoulder or forelimb of the animal and taking periodic video recordings of the movement for analysis, (ii) running backscatter to assess bidirectional communication and/or measure impedance, and (iii) running a therapy paradigm analogous to iTBS stimulation.

#### Video analysis

In post-analysis, the audio cues were used to find the approximate timing of stimulation pulses. The videos were then analyzed with DeepLabCut by marking a stationary point in the scene and features on the area of the pig that was moving during stimulation. The distance between the stationary point and the moving point was measured over time, and the signal was high-pass–filtered above 1 Hz since the response to stimulation was at 2 Hz. Peaks were then identified in the response within a 350-ms period following the application of each stimulation pulse (5 pulses for each trial). The peak values were extracted across each stimulation level applied. Because the angle and distance of the recorded video changed each day, the relative amplitudes of movement are difficult to compare, so the resulting values are normalized to a 0 to 1 scale based on the minimum and maximum peaks recorded that day. Statistical significance between 7-V stimulation and 14-V stimulation is evaluated with an independent sample *t* test (*P* < 0.05).

#### Impedance measurement and evaluation

Output impedance is measured using the onboard circuitry during stimulation (see the “PCBA design” section). These data were transmitted via uplink during chronic testing, with each impedance measurement preceded by a calibration message to ensure proper alignment and calculate the threshold between 0 and 1 data. The transmitter was programmed to trigger the device to apply a 500-Hz stimulation burst at 7-, 8-, and 9-V biphasic amplitudes sequentially and measure the impedance at each level. The uplink data were logged in a text file with the envelope detector values sampled during each bit of each message. In post-analysis, measurement sequences with successful calibration at each of 7, 8, and 9 V were extracted, and the average impedance measured across these three values is reported as the impedance value.

#### Surgical explantation

Animals were prepared similarly to implantation by veterinary staff. Before removing the device in the third cohort, a similar procedure was completed where stimulation was driven by the DOT, and EMG recordings were captured via the Cadwell IOMAX system with subsequent motor output.

A similar 4 cm by 5 cm window was prepared to gain access to the previously implanted device. Using typical neurosurgical instrumentation, the devices were explanted, the incision cleaned and closed using a stapler, and the animals went into recovery. Recovery consisted of the same protocol done following implantation.

#### Terminal Procedure and Euthanasia

Animals were prepared for sacrifice approximately 1 week after explantation. Pigs were put under heavy sedation and then euthanized using a combination of Telazol, Glycopyrrolate, and Somnasol.

#### Tissue Harvesting and Histology

After euthanasia, the brains were removed for histological examination. The brain specimens were subjected to 10% buffered formalin solution for a minimum duration of 7 days. Subsequently, a meticulous brain dissection procedure was carried out to isolate the pertinent brain regions for detailed microscopic examination. For histological assessment, formalin-fixed paraffin-embedded sections with a thickness of 5 μm were prepared. Hematoxylin and eosin staining and immunohistochemistry staining techniques were then applied to these sections. Specifically, ionizing calcium adapter binding molecule 1 and glial fibrillary acidic protein were used as markers to discern and evaluate microglial and astrocyte reactivity and activation, respectively.
