# Supplementary material for: Miniature battery-free epidural cortical stimulators
Source: Sci Adv. 2024 Apr 12;10(15):eadn0858. doi: 10.1126/sciadv.adn0858 (PMC11014439; doi:10.1126/sciadv.adn0858)
Supplement: Supplementary file 1 — Figs. S1 to S4 Table S1 Legends for movies S1 and S2 [file sciadv.adn0858_sm.pdf]

Science Advances
AAAS

Supplementary Materials for

Miniature battery-free epidural cortical stimulators

Joshua E. Woods *et al.*

Corresponding author: Jacob T. Robinson, jtrobinson@rice.edu

Sci. Adv. **10**, eadn0858 (2024)
DOI: 10.1126/sciadv.adn0858

The PDF file includes:

Figs. S1 to S4
Table S1
Legends for movies S1 and S2

Other Supplementary Material for this manuscript includes the following:

Movies S1 and S2

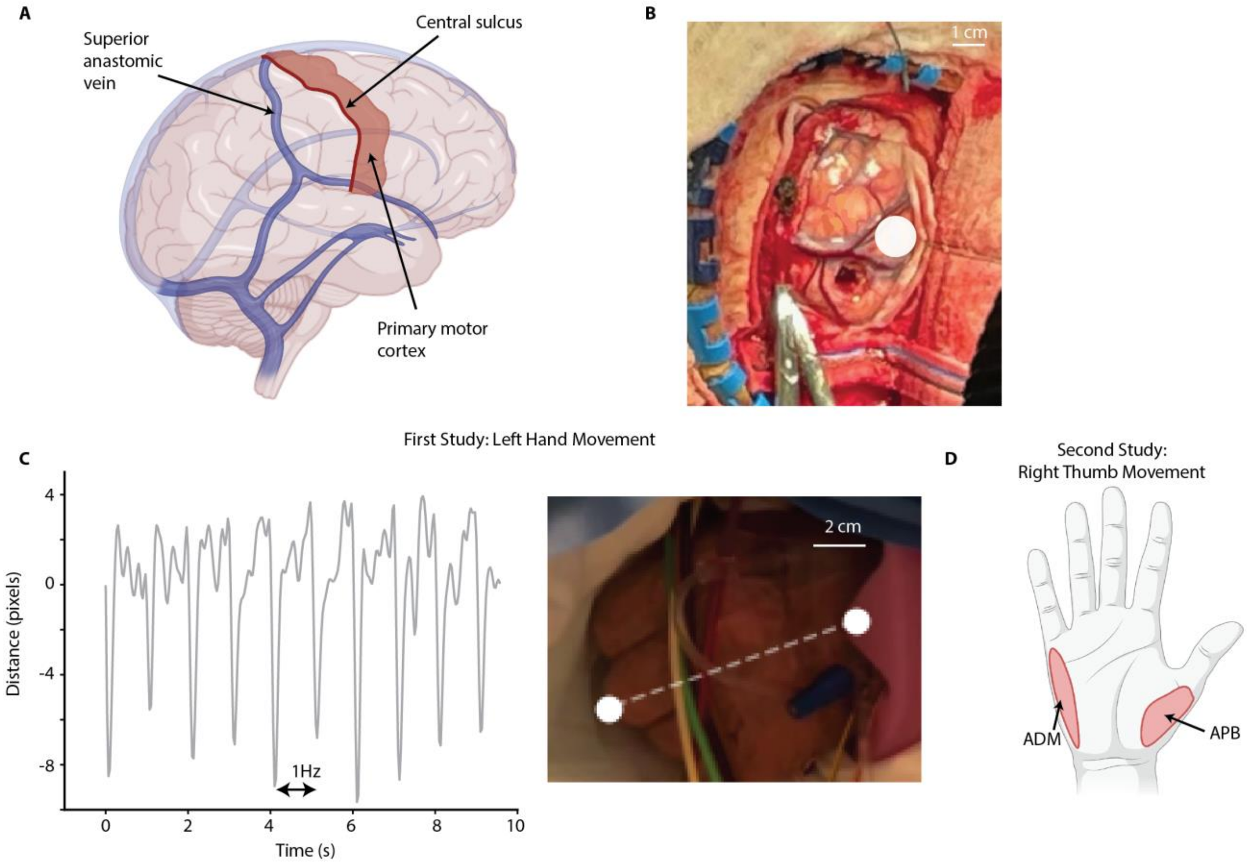

Fig. S1. Intraoperative human supplemental schematics and data. (**A**) Anatomy of the right brain hemisphere showing relevant anatomical markers and the location of the primary motor cortex where stimulation was applied. (**B**) Photograph of the surgical site for the first intraoperative patient with the location of the DOT on the motor cortex denoted with a white circle. (**C**) Results from the first intraoperative human study show movement of the fist clenching in the left hand. Video analysis shows this movement is consistent with the 1 Hz stimulation applied by the DOT. (**D**) Schematic showing the location of the muscles in the right hand where results from the second intraoperative study (Main text, Fig. 1) recorded EMG activity (ADM-APB electrode channels). The location of these muscles is consistent with the thumb movement recorded visually.

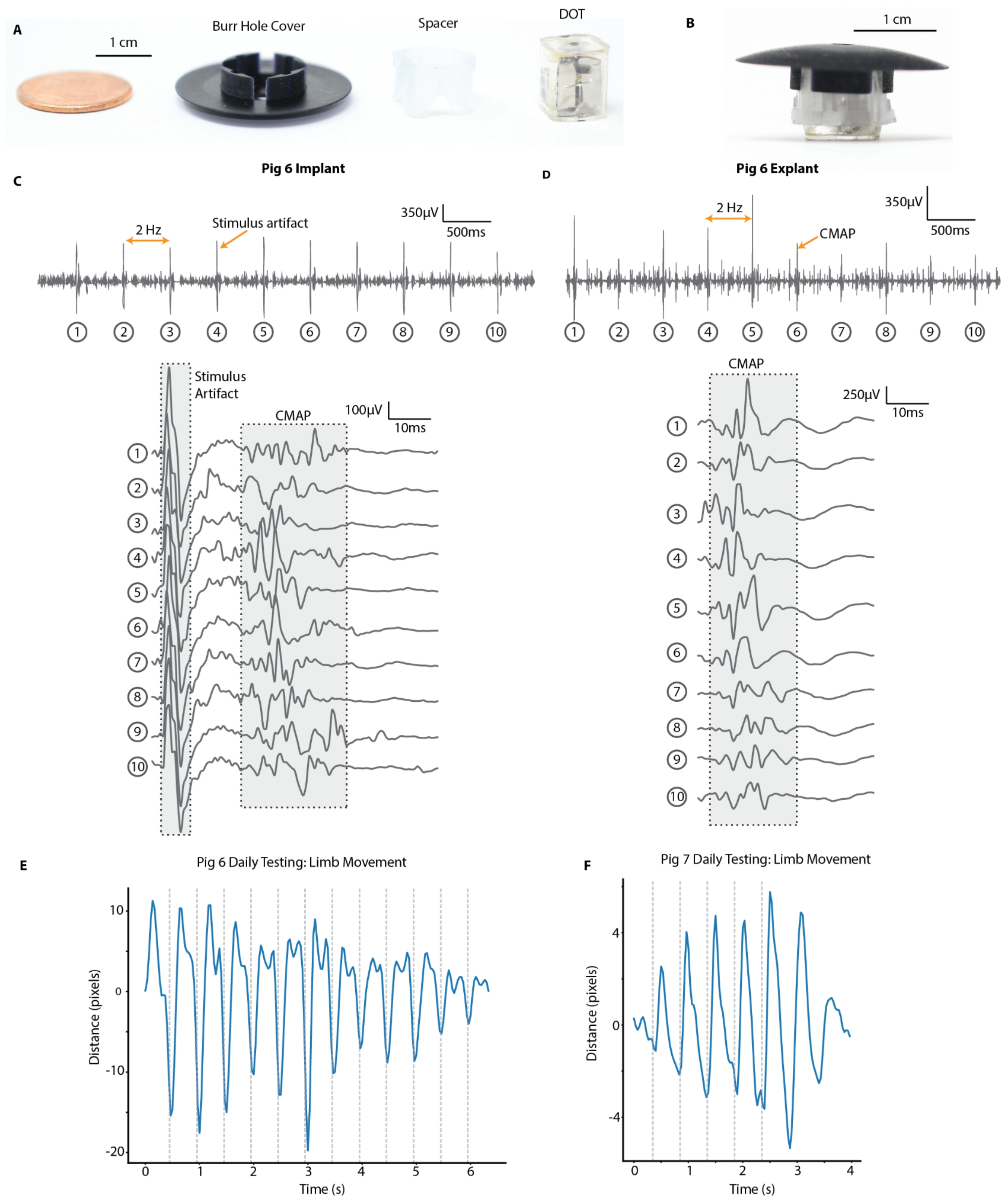

Fig. S2. Additional in vivo porcine results. (**A**) The parts of the implant assembly-including the burr hole cover to protect the implant from impact, the spacer to fill in the rest of the 14 mm burr hole and the DOT-unassembled with a 1c coin and (**B**) assembled for implantation. (**C**) EMG recordings of muscle activity in the right leg of pig 6 activated by DOT stimulation of the motor cortex taken during implantation, and (**D**) explantation verifying chronic functionality. (**E**) Video analysis example of motor movement in the right side in freely behaving animals in Pig 6 and (**F**) Pig 7 demonstrating chronic stimulation functionality across multiple animals.

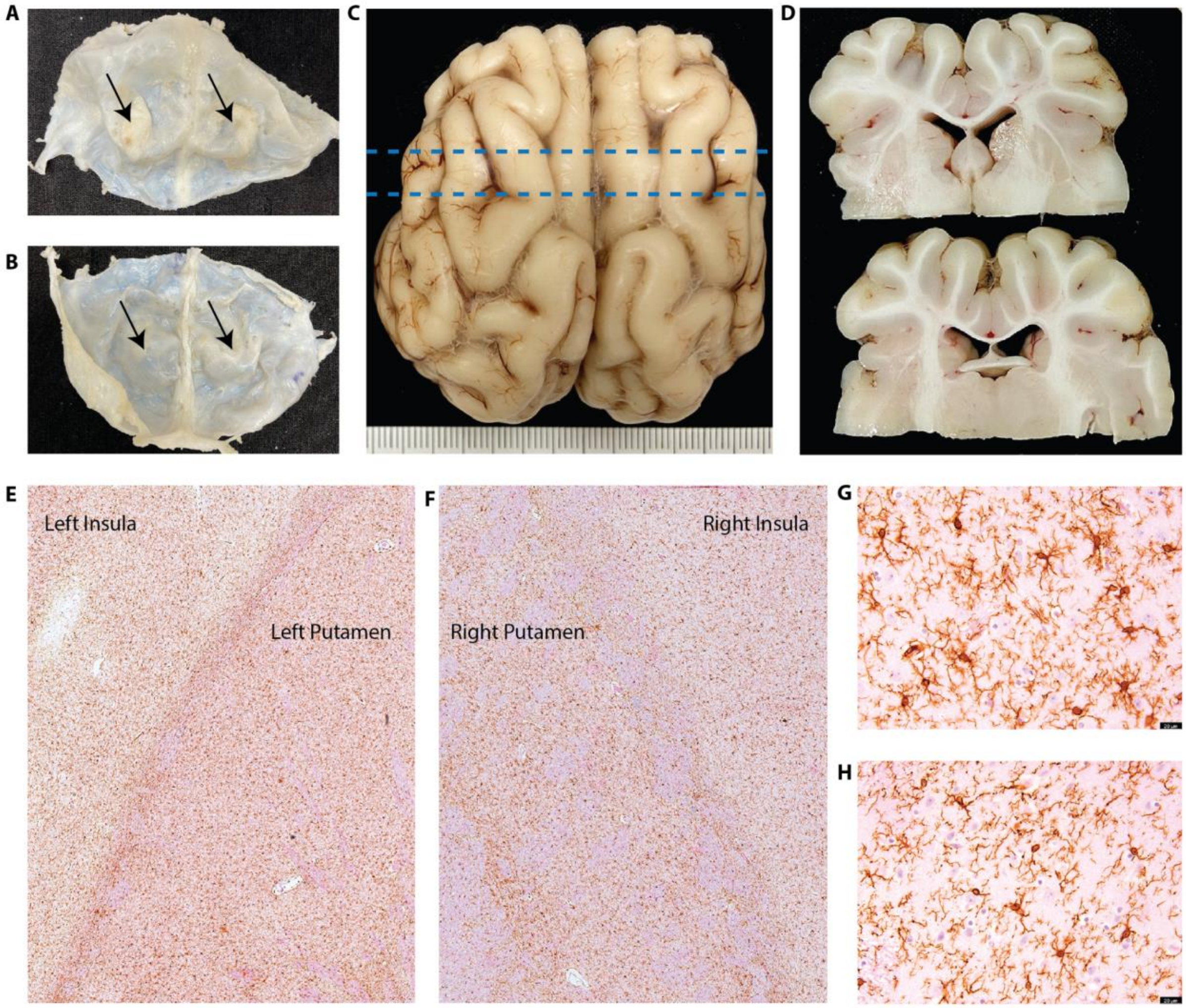

Fig. S3. Porcine brain histology results show no signs of pathology or damage to brain tissue in bilateral implantation comparison. (**A**) Top and (**B**) bottom of the dura show thickening on the top of the dura where the implant (left) and sham (right) were located (denoted with arrows) (**C**) Convexity view of the brain shows no gross cortical damage, blue lines indicate the locations of the views of the slice shown in (**D**) which again shows no tissue damage. Iba1 immunostaining for microglia shows expression on both the left (**E,G**) and right (**F,H**) sides with slightly increased number of microglia in the left side which can be expected as a result of stimulation.

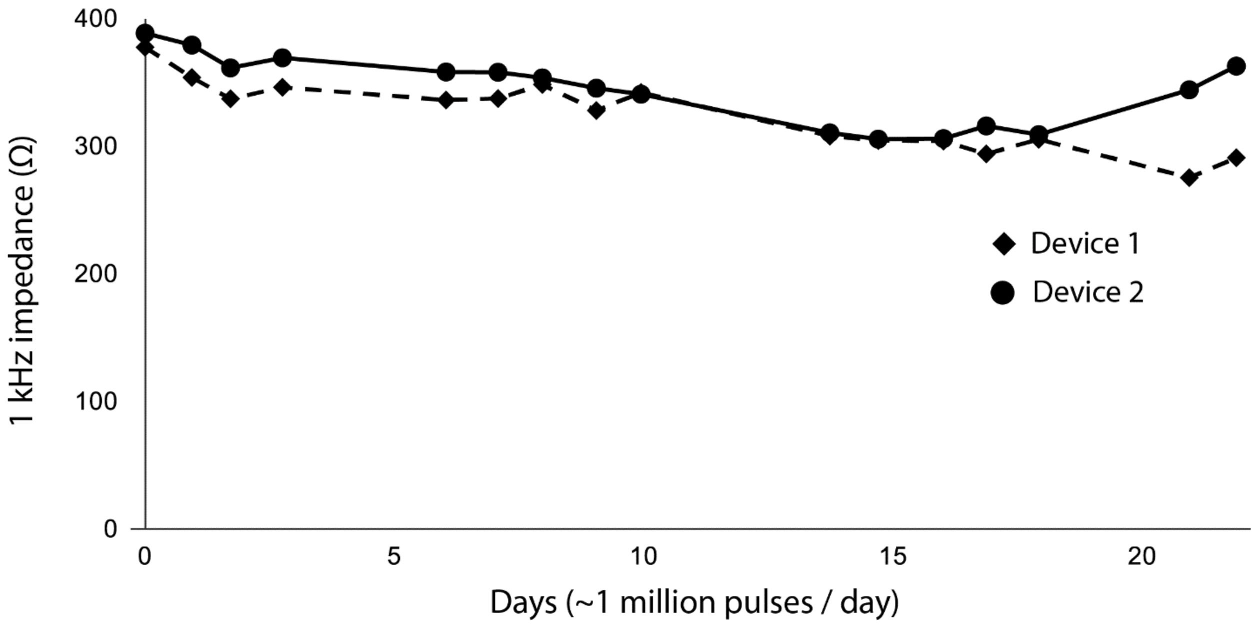

Fig. S4. Durability test of two devices with IrOx electrodes. The devices were connected to a stimulator programmed to produce 250 μs pulse width, 500 Hz, 6 pulse, trains at 2 Hz frequency and 15 V biphasic amplitude, corresponding to roughly 1 million pulses per day. The impedance between the two contacts in saline at 1 kHz was measured daily for 22 days. Over the entire experiment, corresponding to more than 22 million pulses, the devices maintained an impedance between 250 Ω and 400 Ω.

Cohort	Pig/Implant Side	Minutes of stim	Days implanted	Dura thickening	Focal brain tissue loss	White matter microglia	Infection at surgical site	Implant damaged
1	Pig 1/L	Sham	21	Y	N	N	N	N
	Pig 2/L	Sham	21	X	X	X	N	Y*
2	Pig 3/L	Sham	18	X	X	X	N	N
	Pig 4/L	Sham	18	X	X	X	N	N
3	Pig 5/L	Sham	20	Y	Y	Y	N	Y**
	Pig 6/L	170	20	X	Y	Y	N	N
4	Pig 7/R	Sham	35	Y	N	Y	N	N
	Pig 7/L	200	35	Y	N	Y	Y	N
	**Pig 8/R**	**Sham**	**35**	**Y**	**N**	**Y**	**N**	**N**
	**Pig 8/L**	**300**	**35**	**Y**	**N**	**Y**	**N**	**N**

Y = Yes, N = No, X = No results *No burr hole cover – broken glass, **Epoxy failure

Table S1. Porcine Cohort Summary. Summary of the four porcine cohorts used in this work indicating the implant location, how much stimulation was applied, histology notes, and any adverse events. Bold text denotes the animal used in the brain analysis in Supplementary Fig. 3 and main text Fig. 5.

Movie S1. Left hand movement in response to subdural XCS. In the first intraoperative study the DOT placed directly on the right motor cortex and activated at 1 Hz activated left hand clenching at 1 Hz.

Movie S2. Right hand movement in response to epidural XCS. In the second intraoperative study the DOT placed on the dura above the left motor cortex and activated at 1 Hz activated thumb movement in the right hand at 1 Hz.
